# Trombocitopenia induzida por heparina em paciente com oclusão arterial aguda

**DOI:** 10.1590/1677-5449.004215

**Published:** 2016

**Authors:** Rafael Elias Farres Pimenta, Winston Bonetti Yoshida, Hamilton Almeida Rollo, Marcone Lima Sobreira, Matheus Bertanha, Jamil Victor de Oliveira Mariúba, Rodrigo Gibin Jaldin, Paula Angeleli Bueno de Camargo

**Affiliations:** 1 Universidade Estadual Paulista – UNESP, Faculdade de Medicina de Botucatu, Botucatu, SP, Brasil.

**Keywords:** trombocitopenia, heparina, trombofilia, diagnóstico

## Abstract

A trombocitopenia induzida por heparina é uma complicação grave da terapêutica anticoagulante com heparina e está associada à formação de anticorpos antifator IV plaquetário. Costuma surgir a partir do quinto dia do tratamento, com queda de pelo menos 50% da contagem plaquetária. Em decorrência da ativação plaquetária concomitante, pode ocorrer quadro de trombose, venosa ou arterial, com repercussões clínicas graves. Apresentamos um caso de paciente portador de síndrome do anticorpo antifosfolípide, com quadro de oclusão arterial aguda, que foi tratado cirurgicamente e recebeu heparina não fracionada no intra e pós-operatório. No quinto dia de tratamento anticoagulante, apresentou queda maior de 50% da contagem de plaquetas em relação à contagem pré-heparina. A suspeita de trombocitopenia induzida por heparina e seus aspectos diagnósticos e terapêuticos serão abordados neste desafio terapêutico.

## INTRODUÇÃO

A trombocitopenia transitória após injeção intravascular de heparina não fracionada (HNF) foi inicialmente descrita por Copley e Robb em estudos experimentais com cães, em 1942^1^. O desenvolvimento de complicações trombóticas em pacientes recebendo terapia com HNF foi primeiramente descrito em 1958^2^. A associação entre o desenvolvimento de trombocitopenia e a ocorrência de evento tromboembólico em pacientes recebendo terapia com HNF foi relatada no início da década de 1970^3^. Estudos subsequentes demonstraram que a incidência de trombocitopenia induzida por heparina (TIH) era menor que a encontrada em estudos anteriores, e foi então reconhecido que a heparina poderia causar uma diminuição na contagem plaquetária por dois mecanismos^4^.

A TIH é uma síndrome imuno-hematológica que cursa com ativação plaquetária na presença de heparina, induzindo à sua agregação e podendo provocar graves complicações trombóticas. A frequência de TIH nos pacientes que recebem heparina por mais de 5 dias é de 1 a 6%^5^, com maior probabilidade de ocorrer com a HNF quando comparada com a heparina de baixo peso molecular (HBPM), pois apresenta maior comprimento da cadeia polissacarídea e maior nível de sulfatação da heparina bovina. A TIH é classificada em tipo I e tipo II^6^.

A TIH tipo I, uma trombocitopenia não imune associada à heparina, é a forma mais frequente, podendo ocorrer em até 30% dos pacientes. Caracteriza-se por uma supressão não imunológica, benigna, transitória e moderada, da produção e do número de plaquetas. O diagnóstico clínico e laboratorial é definido nos 2 primeiros dias após o início da terapia com heparina, quando ocorre uma moderada trombocitopenia. Raramente a contagem plaquetária é inferior a 100.000 mm^3^ 7. O mecanismo da TIH tipo I está provavelmente relacionado ao efeito pró-agregação plaquetária, o que resulta no aumento de sequestro de plaquetas pelo baço e, portanto, na trombocitopenia. A queda na contagem plaquetária não apresenta significância clínica, e o número de plaquetas pode se normalizar, mesmo que a administração da heparina seja mantida^8^.

A TIH tipo II, também denominada trombocitopenia imunológica induzida por heparina, é uma síndrome imuno-hematológica mediada por um anticorpo que causa ativação plaquetária na presença de heparina e induz à agregação plaquetária. Após a primeira exposição à heparina, entre o quinto e o 14º dia de terapia, a contagem plaquetária pode sofrer redução igual ou superior a 50% em relação à contagem plaquetária pré-heparina (geralmente inferior a 100.000/mm^3^) e pode estar associada a graves complicações trombóticas, com chance de levar à morte^9^.

A suspeita de TIH e seus aspectos diagnósticos e terapêuticos serão abordados neste desafio terapêutico.

## PARTE I: A SITUAÇÃO

**Figura 1:**
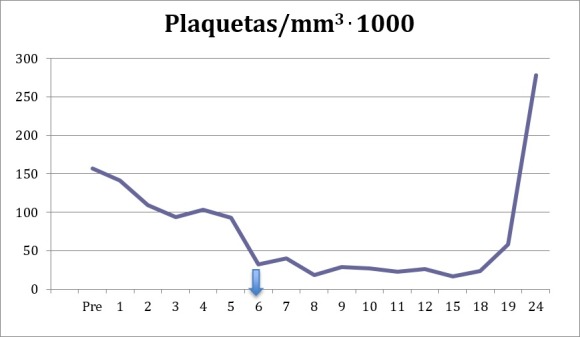
Contagem pré-operatória de plaquetas e ao longo do período pós-operatório, com seta no momento da suspensão da heparinoterapia.

Paciente de 40 anos, sexo masculino, branco, deu entrada no pronto-socorro com quadro clínico de dor, esfriamento, palidez e ausência de pulsos poplíteo e distais no membro inferior direito (MID) havia quatro dias. Foi feito então o diagnóstico de oclusão arterial aguda (OAA), classificação IIa de Rutherford. Negava antecedentes de claudicação intermitente, flebites, tromboses, diabetes melito, hipertensão arterial sistêmica, acidente vascular encefálico isquêmico, infarto agudo do miocárdio e arritmia cardíaca. Era tabagista e tinha passado de alcoolismo. O exame ultrassonográfico vascular mostrou imagem compatível com oclusão da transição do ramo superficial da artéria femoral comum com a artéria poplítea. Foi operado de emergência pela técnica de tromboembolectomia com cateter de Fogarty, seguida de angioplastia do ramo superficial da artéria femoral comum e fasciotomia do compartimento posterior da perna direita. Recebeu HNF no intraoperatório (5.000 UI endovenosa em *bolus*) e pós-operatório (dose inicial de 30.000 UI/dia endovenosa, ajustada posteriormente para manutenção do tempo de tromboplastina parcial ativada entre 1,5 e 2,5). A contagem pré-operatória de plaquetas foi de 157.000/mm^3^ (Figura 1). Foi reoperado para tentativa de restauração vascular outras duas vezes, em 24 e 48h, em decorrência de retrombose arterial no mesmo sítio.

A partir do terceiro dia de tratamento com HNF, começou a apresentar queda na contagem de plaquetas (109.000/mm^3^), que progrediu até 32.000/mm^3^ no sexto dia de pós-operatório. Foi então suspensa a administração da HNF (Figura 1), devido à suspeita de TIH (queda > 50% na contagem de plaquetas a partir do quinto dia de HNF) (Figura 1).

## PARTE II: O QUE FOI FEITO

Devido às complicações da isquemia, no sétimo dia de pós-operatório o paciente foi submetido a amputação aberta em guilhotina em nível do tornozelo do MID. Pela idade e ausência de outros fatores de risco para aterosclerose, foi feita a hipótese clínica de arterite para etiologia da isquemia arterial aguda, e exames laboratoriais para a investigação de colagenoses foram solicitados. Anticorpos antinucleares (anti-ENA = antígenos nucleares extraíveis pela saliva; subtipos anti-snRNP = *small nuclear ribonucleoproteins*; SM = anti-Smith; dsDNA = anti-double stranded DNA), pesquisa de células LE e prova do látex foram normais. Estavam alterados velocidade de hemossedimentação (32 mm/h), PCRt (7,8 mg/dl), fibrinogênio (579 mg/dl), alfa 1-glicoproteína ácida (169 mg/dl) e fator antinuclear (1/5120). O exame anatomopatológico do produto de amputação nada revelou além de trombose de artéria tibial posterior e necrose de músculos. No 16º dia de internação, foi submetido a amputação fechada do MID em terço proximal de perna. Recuperou-se bem e recebeu alta no 30º dia de internação. No seguimento após a alta, foram feitos exames de trombofilia, como dosagem de antitrombina, proteínas C e S, anticoagulante lúpico e anticardiolipina IgM, que foram normais. Os anticorpos anticardiolipina IgG apresentaram níveis elevados (120 GPL/Uml, valor de referência até 40 GPL/Uml). Essa elevação da anticardiolipina IgG manteve-se em nova dosagem 12 semanas depois, com valor de 60 GPL/Uml, o que confirmou o diagnóstico de síndrome do anticorpo antifosfolípide (SAF). Houve o preenchimento de um critério clínico (trombose arterial) e um critério laboratorial (anticardiolipina IgG > 40 GPL/Uml) em duas medidas, com intervalo de pelo menos 12 semanas entre elas, para esse diagnóstico. Não foi possível confirmar laboratorialmente a TIH por falta de dosagem de anticorpos para TIH no hospital, mas a suspeita clínica permaneceu forte. Pelo fato de o paciente ser portador de SAF e ter apresentado uma trombose arterial, optamos por anticoagulá-lo com varfarina perenemente.

Conforme mostrado na Figura 1, com a suspensão da heparina, os níveis de plaquetas voltaram ao normal após o 24º dia de pós-operatório.

## DISCUSSÃO

A TIH tipo II é uma doença imunomediada rara e costuma ser grave, provocando trombocitopenia após 5 a 15 dias do início da heparinoterapia. Paradoxalmente, tem risco alto de complicações tromboembólicas^10^. A TIH tipo II ocorre em 1 a 6% dos pacientes tratados com HNF e em até 0,9% dos pacientes tratados com HBPM. Entre 33 e 50% desses casos cursam com tromboses venosas ou arteriais e com risco alto de amputações^11^.

A etiologia da TIH tipo II é a formação de anticorpos tipo IgG contra o complexo heparina e o fator IV plaquetário, que são então identificados como antígeno^12^. Os imunocomplexos reagem com o receptor FcyRIIA das plaquetas^13^ e as ativam, levando à sua agregação e também à liberação de maior quantidade de fator plaquetário. Isso culmina na ativação da trombina e da cascata da coagulação, que favorece a formação de trombos e a destruição das plaquetas^10^^,^ 11.

Recomenda-se que todo paciente em heparinoterapia tenha uma contagem prévia de plaquetas no início do tratamento, que seja repetida a cada 2 dias. Constatando-se queda de mais de 50% no número de plaquetas a partir do quinto dia de tratamento, deve-se suspeitar de TIH e suspender imediatamente a heparina^11^^,^ 14. O diagnóstico pode ser confirmado pelo método funcional, que mede a ativação plaquetária causada pelo anticorpo heparina dependente *in vitro* 11, mas ele não está rotineiramente disponível em todos os hospitais^13^. Existem escores de risco para fortalecer a suspeita diagnóstica (4T ou HEP *score*), porém de pouca utilidade clínica, devido à baixa sensibilidade e especificidade^13^.

No Brasil, a droga para tratamento dos pacientes com TIH tipo II é a fondaparinux^15^. É possível que a rivaroxabana possa ser uma alternativa, embora não haja estudos com essa droga para esse fim. Em outros países, são recomendados argatroban, hirudina, bivalirudina e danaparoid^13^. Deve-se evitar a introdução precoce da varfarina como alternativa, uma vez que há risco de aumento do estado pró-trombótico, devido à rápida redução dos níveis séricos de proteína C, um anticoagulante natural. Porém, tão logo os níveis plaquetários voltem ao normal, pode-se introduzi-la com doses menores que 5 mg/dia e, em seguida, com doses reajustadas, mantendo a razão normalizada internacional (RNI) entre 2,0 e 3,0^13^.

Nosso paciente tinha como comorbidade a SAF, uma trombofilia adquirida que pode provocar tromboses arteriais (30%) ou venosas (70%), trombocitopenia e complicações obstétricas, sendo, portanto, um diagnóstico diferencial importante a ser considerado para o caso relatado. A prevalência do anticorpo antifosfolípide em pacientes com lúpus eritematoso sistêmico (LES) é de aproximadamente 40%, e as manifestações clínicas da SAF estão presentes, provavelmente, em 30 a 40% dos pacientes que possuem tal anticorpo ou em cerca de 10 a 15% dos pacientes lúpicos^16^. Foi identificado somente um critério para LES em nosso caso (fator antinuclear positivo), porém sem completar os demais critérios mínimos para fechar o diagnóstico. Por ser uma trombofilia com alta incidência de recidiva, recomenda-se anticoagulação por 12 meses após o primeiro episódio de trombose venosa e perene após o segundo episódio ou após trombose arterial^17^.

No caso tratado, o quadro de TIH foi bastante típico, pois a redução de mais de 50% na contagem plaquetária ocorreu entre o quinto e o sexto dias de heparinoterapia. Além disso, foi transitório, regredindo com a suspensão da HNF. Infelizmente, os testes de confirmação não estão disponíveis em nosso serviço, o que nos impede de ter segurança absoluta no diagnóstico.

## CONCLUSÕES

A TIH não é uma complicação muito frequente, mas deve sempre ser lembrada, com estabelecimento de rotina de contagens plaquetárias antes e a cada dois dias depois do tratamento anticoagulante com heparinas.
