# Supplementary material for: Trombocitopenia induzida por heparina em paciente com oclusão arterial aguda
Source: J Vasc Bras. 2016 Apr-Jun;15(2):138–41. [Article in Portuguese] doi: 10.1590/1677-5449.004215 (PMC5829708; doi:10.1590/1677-5449.004215)
Supplement: Translation

# Translation: Heparin induced thrombocytopenia in a patient with acute arterial occlusion

**Authors:** Rafael Elias Farres Pimenta, Winston Bonetti Yoshida, Hamilton Almeida Rollo, Marcone Lima Sobreira, Matheus Bertanha, Jamil Victor de Oliveira Mariúba, Rodrigo Gibin Jaldin, Paula Angeleli Bueno de Camargo

**Affiliations:** 1 Universidade Estadual Paulista – UNESP, Faculdade de Medicina de Botucatu, Botucatu, SP, Brazil.

**Keywords:** thrombocytopenia, heparin, thrombophilia, diagnosis and therapy

## Abstract

Heparin induced thrombocytopenia (HIT) is a serious complication of heparin anticoagulation and is associated with formation of anti-platelet factor 4. It usually occurs from the fifth day of treatment onwards, with a fall in platelet count of at least 50%. Venous or arterial thrombosis may occur as a result of concomitant platelet activation, with serious clinical repercussions. We present the case of a patient with antiphospholipid antibody syndrome who presented with acute arterial occlusion and was treated surgically and given unfractionated heparin intraoperatively and postoperatively. On the fifth day of anticoagulant treatment he exhibited a platelet count decreased by more than 50% compared to the count prior to heparin administration. The suspicion of heparin-induced thrombocytopenia and its diagnostic and therapeutic features are addressed in this therapeutic challenge paper.

## INTRODUCTION

Transitory thrombocytopenia after intravascular injection of unfractionated heparin (UFH) was first described by Copley and Robb in experimental studies with dogs, in 1942.^1^ Development of thrombotic complications in patients given UFH treatment was first described in 1958.^2^ The association between developing thrombocytopenia and occurrence of a thromboembolic event in patients being treated with UFH was reported at the start of the 1970s.^3^ Later studies demonstrated that the incidence of heparin-induced thrombocytopenia (HIT) was lower than observed in previous studies and that was when it was recognized that heparin can cause reduced platelet counts via two mechanisms.^4^

Heparin-induced thrombocytopenia is an immunohematological syndrome that involves platelet activation in the presence of heparin, causing them to aggregate, and it can cause severe thrombotic complications. The frequency of HIT among patients given heparin for more than 5 days is from 1 to 6%,^5^ with a higher probability of occurrence with UFH compared with low molecular weight heparin (LMWH), because it has a longer polysaccharide chain and higher level of sulfation from the bovine heparin. Heparin-induced thrombocytopenia is classified as type I or type II.^6^

Type I HIT, a nonimmune heparin-associated thrombocytopenia, is the more common form and can occur in up to 30% of patients. It is characterized by non-immunological, benign, transitory and moderate suppression of platelet production and numbers. Clinical and laboratory diagnosis is made within the first 2 days after starting treatment with heparin, when moderate thrombocytopenia sets in. Platelet counts rarely drop below 100,000 mm^3^.^7^ The mechanism of Type I HIT is probably related to the platelet pro-aggregating effect, which results in increased platelet sequestration by the spleen and, as a result, in thrombocytopenia. The fall in platelet count does not have clinical significance and the number of platelets may normalize even if administration of heparin is maintained.^8^

Type II HIT, also known as heparin-induced immunological thrombocytopenia, is an immunohematological syndrome mediated by an antibody that causes platelet activation in the presence of heparin and induces platelet aggregation. After the first exposure to heparin, between the fifth and fourteenth days of treatment, the platelet count can undergo a reduction greater than or equal to 50% in relation to the pre-heparin platelet count (generally lower than 100,000/mm^3^) and this type can be associated with severe thrombotic complications, with a risk of death.^9^

The suspicion of heparin-induced thrombocytopenia and its diagnostic and therapeutic features are addressed in this therapeutic challenge.

## PART I: CLINICAL SITUATION

A 40-year-old, white, male patient was admitted to the emergency room with a clinical status of pain, coldness, pallor and absent popliteal and distal pulses in the right lower limb (RLL), with onset 4 days previously. He was diagnosed with acute arterial occlusion (AAO), with a IIa Rutherford classification. He denied any history of intermittent claudication, phlebitis, thromboses, diabetes mellitus, systemic arterial hypertension, ischemic cerebrovascular accident, acute myocardial infarction or cardiac arrhythmia. He was a smoker and had been alcoholic in the past. Vascular ultrasonography showed images compatible with occlusion of the transition from the superficial branch of the common femoral artery to the popliteal artery. He underwent emergency thromboembolectomy with a fogarty catheter technique, followed by angioplasty of the superficial branch of the common femoral artery and fasciotomy of the posterior compartment of the right leg. He was given UFH intraoperatively (5,000 UI intravenously in bolus) and postoperatively (initial dose of 30,000 UI/day intravenously, with later adjustment to maintain activated partial thromboplastin time between 1.5 and 2.5). His preoperative platelet count was 157,000/mm^3^ (Figure 1). He was reoperated twice more for attempts at vascular repair, at 24 and 48h, because of repeat arterial thrombosis at the same site.

**Figure 1:** Preoperative and postoperative platelet counts, with arrow indicating when heparin was withdrawn.

Starting on the third day of treatment with UFH, his platelet count began to fall (109,000/mm^3^), continuing to drop until it reached 32,000/mm^3^ on the sixth postoperative day. Administration of UFH was then suspended (Figure 1), due to a suspicion of HIT (> 50% drop in platelet count after five days of UFH) (Figure 1).

## PART II: WHAT WAS DONE

In response to complications of ischemia, on the seventh postoperative day, the patient underwent an RLL open guillotine amputation at the level of the ankle. In view of the patient’s age and absence of other risk factors for atherosclerosis, a clinical hypothesis of arteritis was suspected in response to the etiology of acute arterial ischemia, and so laboratory tests were requested for investigation of connective tissue diseases. Test results were normal for antinuclear antibodies (anti-ENA = saliva extractable nuclear antigens; anti-snRNP subtypes = small nuclear ribonucleoproteins; SM = anti-Smith; dsDNA = anti-double stranded DNA), LE cells and latex fixation test. Results were abnormal for erythrocyte sedimentation rate (32 mm/h), titrated CRP (7.8 mg/dl), fibrinogen (579 mg/dl), alpha 1-acid glycoprotein (169 mg/dl) and antinuclear factor (1/5120). Pathology results for the amputation specimen did not reveal anything other than thrombosis of the posterior tibial artery and necrosis of muscles. Sixteen days after admission, the patient underwent an RLL closed amputation at the proximal third of the leg. He recovered well and was discharged 30 days after admission. During post-discharge follow-up, tests for thrombophilia were conducted, including antithrombin, C and S proteins, lupus anticoagulant and IgM anticardiolipin assays, all of which were normal. Anticardiolipin IgG antibody levels were elevated (120 GPL/Uml, reference value is ≤ 40 GPL/Uml). Anticardiolipin IgG remained elevated when the assay was repeated 12 weeks later, at 60 GPL/Uml, which confirmed the diagnosis of antiphospholipid antibody syndrome (AAS). One clinical criterion supporting this diagnosis was met (arterial thrombosis) and one laboratory criterion supporting this diagnosis was met (anticardiolipin IgG > 40 GPL/Uml in two assays, with an interval of at least 12 weeks). It was not possible to confirm HIT on the basis of test results because HIT antibodies had not been assayed at the hospital, but the clinical suspicion remained strong. Since the patient has AAS and had already had arterial thrombosis, we chose to put him on permanent anticoagulation with warfarin.

As shown in Figure 1, the platelet count rose when heparin was withdrawn, returning to normal 24 days after the operation.

## DISCUSSION

Type II HIT is rare immunomediated disease that is generally severe, provoking thrombocytopenia from 5 to 15 days after starting heparin treatment. Paradoxically, it involves a high risk of thromboembolic complications.^10^ Type II HIT occurs in 1 to 6% of patients treated with UFH and in up to 0.9% of patients treated with LMWH. From 33 to 50% of these cases progress to venous or arterial thromboses and there is a high risk of amputations.^11^

The etiologic basis of type II HIT is formation of IgG type antibodies against the heparin complex and platelet factor 4, which are identified as antigens.^12^ The imunocomplexes react with platelet FcyRIIA receptors^13^ activating them and provoking aggregation and release of greater quantities of platelet factor. This culminates in activation of thrombin and the coagulation cascade, which stimulates formation of thrombi and destruction of platelets.^10^^,^ 11

It is recommended that all patients to be given heparin therapy should have a baseline platelet count at the start of treatment and the test should be repeated every 2 days. If it is observed that the platelet count has fallen by more than 50% from the fifth day of treatment on, HIT should be suspected and heparin suspended immediately.^11^^,^ 14 Diagnosis can be confirmed by the functional method, which measured platelet activation caused by the heparin-dependent antibody in vitro,^11^ but the test is not routinely available in all hospitals.^13^ Risk scores designed to strengthen diagnostic suspicion exist (4T or HEP score), but they are of little clinical utility because of their low sensitivity and specificity.^13^

In Brazil, fondaparinux is the drug used for treating patients with Type II HIT.^15^ It is possible that rivaroxaban may be an alternative possibility, but there are no studies of use of this drug for this purpose. In other countries, argatroban, hirudin, bivalirudin and danaparoid are recommended.^13^ Premature introduction of warfarin as an alternative should be avoided, since there is a risk of increasing the pro-thrombotic state, because of a rapid reduction in serum levels of C protein, which is a natural anticoagulant. However, as soon as platelet levels return to normal, it can be introduced at doses below 5 mg/day, and then adjusted to maintain the international normalized ratio between 2.0 and 3.0.^13^

Our patient had a comorbidity, AAS, which is an acquired thrombophilia that can provoke arterial (30%) or venous (70%) thromboses, thrombocytopenia and obstetric complications, and so it is an important differential diagnosis to be considered in the case described. The prevalence of the antiphospholipid antibody in patients with systemic lupus erythematosus is approximately 40%, and clinical manifestations of AAS are probably present in 30 to 40% of the patients who have this antibody or in around 10 to 15% of those with lupus.^16^ Just one criterion for systemic lupus erythematosus was identified in our case (positive for antinuclear antibodies), but the patient did not meet the other minimum criteria for this diagnosis. As this is a thrombophilia with a high incidence of relapse, it is recommended that anticoagulation be maintained for 12 months after a first episode of venous thrombosis and indefinitely after a second episode or arterial thrombosis.^17^

The presentation of HIT in this case was highly typical, since the platelet count reduction by more than 50% occurred on the fifth and sixth days of treatment with heparin. The condition was also transitory, regressing after withdrawal of UFH. Unfortunately, the tests needed for confirmation are not available at our service, which prevents us from being absolutely certain of the diagnosis.

## CONCLUSIONS

Heparin-induced thrombocytopenia is an uncommon complication, but it should always be remembered, and it should be made routine to take a platelet count before and every 2 days after starting anticoagulant treatment with heparin.
